# Supplementary material for: Prevalence and consequences of non-adherence to an evidence-based approach for incidental pulmonary nodules
Source: PLoS One. 2022 Sep 9;17(9):e0274107. doi: 10.1371/journal.pone.0274107 (PMC9462825; doi:10.1371/journal.pone.0274107)
Supplement: S1 Table — (DOCX) [file pone.0274107.s001.docx]

**S1 Table. Invasive procedures for benign processes.**

| Surgery for benign disease (n=7) | | |
| --- | --- | --- |
|  | Sarcoidosis | 3 |
|  | Benign (necrotizing granulomas) | 3 |
|  | Infection | 1 |
| Non-surgical* biopsy for benign disease (n=15) | | |
|  | Sarcoidosis | 6 |
|  | Non-diagnostic | 3 |
|  | Infection | 3 |
|  | Granuloma (non-sarcoid) | 2 |
|  | Rheumatoid nodule | 1 |
| *Non-surgical biopsy includes transthoracic biopsies and transbronchial biopsies | | |
