# Supplementary material for: Prevalence and consequences of non-adherence to an evidence-based approach for incidental pulmonary nodules
Source: PLoS One. 2022 Sep 9;17(9):e0274107. doi: 10.1371/journal.pone.0274107 (PMC9462825; doi:10.1371/journal.pone.0274107)
Supplement: S2 Table — (DOCX) [file pone.0274107.s002.docx]

**S2 Table. Factors associated with an adverse outcome.**

| Predictor variable | | Univariable association | |
| --- | --- | --- | --- |
|  |  | OR | 95% CI |
| Patient factors | | | |
| Age (per decade increase) | | 0.93 | 0.71-1.24 |
| History of tobacco use | | 0.95 | 0.41-2.21 |
| Family history of lung cancer | | 1.01 | 0.33-3.07 |
| Nodule factors | | | |
| Size | | | |
|  | <10 mm | 0.13 | 0.05-0.33 |
|  | 10-15 mm | 2.21 | 0.77-6.32 |
|  | >15 mm | 5.69 | 2.48-13.05 |
| Type of nodule | | | |
|  | Solid | 0.79 | 0.30-2.05 |
|  | Part solid | 2.04 | 0.81-5.15 |
| Upper lobe | | 1.47 | 0.65-3.35 |
| Spiculated | | 3.96 | 1.63-9.57 |
| Pre-test probability of cancer | | | |
|  | Low (<10%) | 0.20 | 0.09-0.46 |
|  | Intermediate (≥10% & <65%) | 4.14 | 1.82-9.43 |
|  | High (≥65%) | 2.55 | 0.68-9.53 |
| Referrals | | | |
|  | Pulmonary | 7.35 | 2.16-25.04 |
|  | Thoracic Surgery | 13.18 | 5.51-31.56 |
| PET scan | | | |
|  | Obtained | 42.86 | 9.86-186.26 |
|  | Negative | 0.68 | 0.09-5.34 |
|  | Intermediate | 13.78 | 4.75-39.97 |
|  | Positive | 8.97 | 3.84-20.98 |
| Adherent to guidelines | | 0.34 | 0.15-0.79 |
| CI: confidence interval; OR: odds ratio; PET: positron emission tomography | | | |
